# Validity evidence for the new ENDO Mentor Suite and its use in the Fundamentals of Endoscopic Surgery examination

**DOI:** 10.1007/s00464-026-12612-z

**Published:** 2026-03-19

**Authors:** Noosha D. Deravi, Payton M. Miller, Maya L. Hunt, Jessica Mischna, Jose M. Martinez, Eric M. Pauli, E. Matthew Ritter

**Affiliations:** 1 Division of General Surgery, Department of Surgery, Indiana University School of Medicine, Indianapolis, IN USA; 2 Society of American Gastrointestinal and Endoscopic Surgeons, Los Angeles, CA USA; 3 Division of General Surgery, DeWitt Daughtry Family Department of Surgery, University of Miami Health System, Miami, FL USA; 4 Division of Minimally Invasive and Bariatric Surgery, Department of Surgery, Penn State College of Medicine, Hershey, PA USA

**Keywords:** Endoscopy, FES, Education, Simulation, Surgery, Assessment

## Abstract

**Background:**

The Fundamentals of Endoscopic Surgery (FES) manual skills exam is a simulation-based assessment originally developed on the GI Mentor II (GIM) with strong validity evidence. Surgical Science has since released the ENDO Mentor Suite (ES), for which validity evidence was previously demonstrated in a small, randomized study. However, there were minor scoring discrepancies for Loop Reduction and Mucosal Inspection. This follow-up study aims to determine if the ES introduces a threat to validity in the form of construct irrelevant variance through analysis of FES examinee performance.

**Methods:**

Retrospective analysis of FES examinee data, including demographics and performance, was conducted following the integration of the ES as a testing platform in 2023. Task and total scores were obtained through a standardized computer-based algorithm. Examinees without self-reported demographics were excluded from analysis. Performance and pass rates were compared between ES and GIM examinees using standard statistical methods and sensitivity analyses within a non-inferiority framework.

**Results:**

Of 1,121 FES examinees, 436 (39%) provided demographic information. Simulator groups were similar in demographics and endoscopy experience. There were small statistically discernable differences in Retroflexion and Tool Targeting scores between ES and GIM examinees. However, total scores (ES 70 ± 12 vs GIM 69 ± 11, *p* = 0.55) and pass rates (ES 88% vs GIM 83%, *p* = 0.25) were similar. Sensitivity analyses also confirmed a similar pattern of non-inferiority.

**Conclusion:**

The ES does not introduce construct irrelevant variance, supporting its use in the FES manual skills exam. This is demonstrated by similar overall performance and pass rates between ES and GIM examinees. Additionally, previously reported variability in Loop Reduction and Mucosal Inspection were not redemonstrated within this larger sample. Given that over half of examinees were excluded from this analysis, measures to improve quality control on demographic reporting will be essential for additional validity investigations.

**Graphical Abstract:**

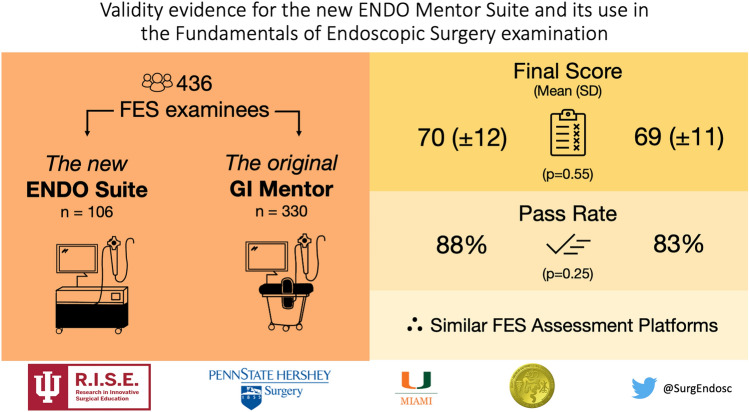

The Fundamentals of Endoscopic Surgery (FES) examination is a cognitive and psychomotor assessment of the knowledge and skills required to safely perform flexible endoscopy [1]. The manual skills portion is a simulation-based assessment consisting of five technical endoscopic tasks. It was originally designed and developed by the Society of American Gastrointestinal and Endoscopic Surgeons (SAGES) on the GI Mentor II (GIM), a virtual reality (VR) simulator by Surgical Science (Fig. 1) [2]. The GIM utilizes an authentic endoscope to navigate through VR modules ranging from basic endoscopic skills to advanced procedures. Multiple studies have demonstrated validity evidence supporting its use in endoscopic skill training and assessment of competency [2, 3]. As such, the GIM has exclusively been used by SAGES as the simulator platform for administration of the FES examination.

**Fig. 1:**
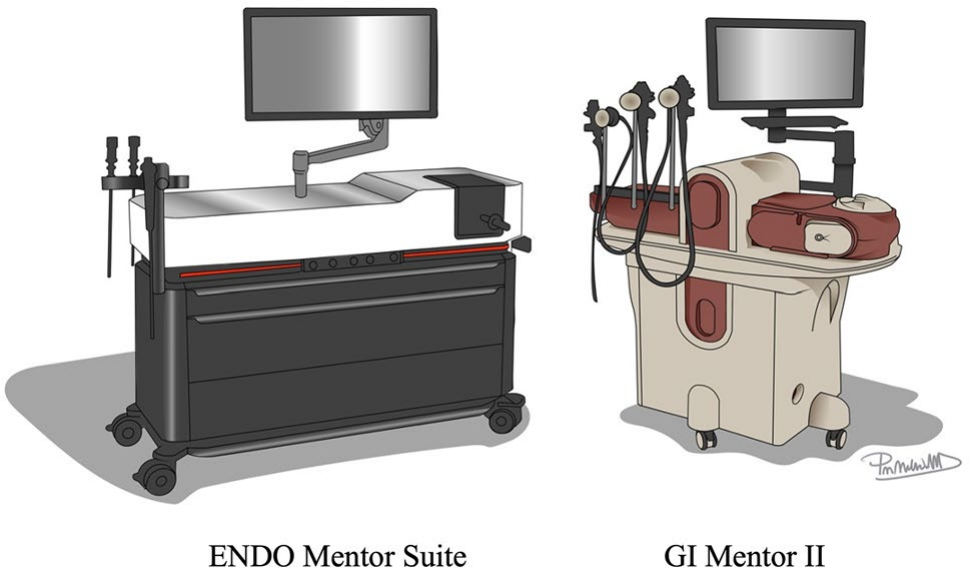
Endoscopic simulator platforms

In 2022, Surgical Science released the ENDO Mentor Suite (ES) platform (Fig. 1). This multi-specialty simulator combines gastrointestinal endoscopy, bronchoscopy, hysteroscopy, and endourology into a single training system [4]. While neither the software nor hardware for the gastrointestinal endoscopy portion was significantly changed, it is still necessary to conduct validity investigations to support the use of this new simulator as it becomes integrated into FES testing centers.

Utilizing the framework for validity as outlined by the *Standards for Educational and Psychological Testing*, preliminary work has provided some initial validity evidence for administration of the FES manual skills examination on the ES through a small, randomized control trial [5, 6]. In this study, surgical trainees and attending surgeons were randomized into two groups to complete the five FES tasks on either (a) the GIM first and the ES second or (b) the ES first and the GIM second. Comparison of scores from the first round demonstrated individual task and overall performance were similar between simulator platforms. However, the second round revealed scoring discrepancies in Loop Reduction and Mucosal Inspection, resulting in statistically discernable differences in mean total scores.

As such, the first round of this preliminary study contributed some relationship to other variables validity evidence in the form of concurrent validity by demonstrating that the ES produces similar performance results to that of a previously established instrument (i.e., the GIM). The scoring discrepancies in the second round, however, raises the question of whether the ES poses a threat to validity. One specific threat to validity is construct irrelevant variance, which is when other variables unrelated to the intended construct of the assessment impacts the observed results. Thus, this follow-up study aims to determine if the ES introduces construct irrelevant variance through analysis of routinely collected FES manual skills examination data that compares performance on the ES to the GIM.

## Methods

**Fig. 2:**
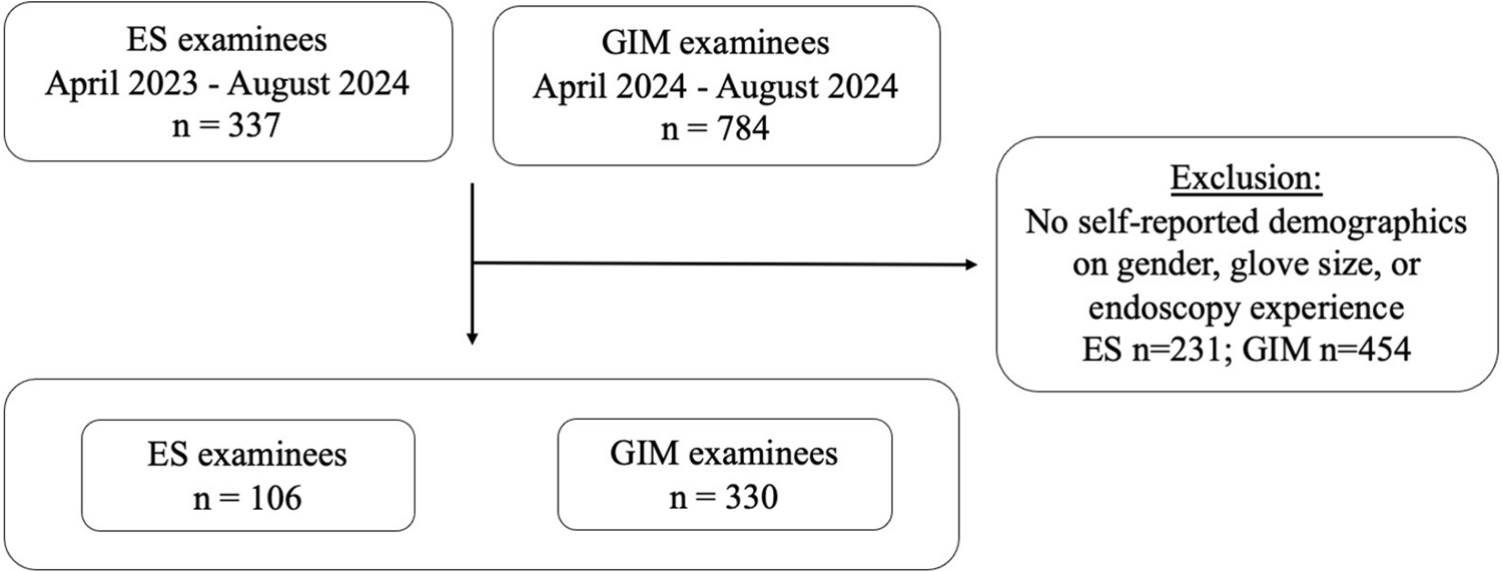
Flow diagram for inclusion of examinees in study

A retrospective analysis was conducted on the FES manual skills examination data following the integration of the ES simulator as a testing platform in 2023. Routinely collected data from the skills exam per SAGES were retrieved, deidentified, and compiled into a secure database. Data from exams administered on the ES between April 2023 and August 2024 were compared to those administered on the GIM platforms, such as GI Mentor II or GI Mentor Express, during an overlapping timeframe (Fig. 2). Analyzed data between simulator platforms included examinee demographics, endoscopy experience, and performance, measured by individual task scores, total score (primary outcome), and pass status (secondary outcome).

### Measures

#### Demographics and experience

All examinees were asked to voluntarily self-report on demographics and endoscopy experience. Demographics included gender, training level, and glove size. Endoscopy experience included number of upper and lower endoscopy cases performed within the last year and overall (categorized as 0–24, 25–49, 50–99, 100–199, or 200+). Many examinees, however, opted out of completing the demographic portion. Thus, a complete-case analysis was conducted and only examinees who self-reported on gender, glove size, and endoscopy experience, were included as prior studies have identified these parameters to have the strongest relationship to FES skills examination performance [7]. To address potential selection bias, overall performance (total score and pass status) of examinees with complete demographic data were compared to those with incomplete demographic data for each simulator platform using two-sample *t* tests and chi-squared test, respectively.

#### Performance scores and pass rates

The FES exam consists of the following five endoscopic tasks: Navigation, Loop Reduction, Retroflexion, Mucosal Evaluation, and Tool Targeting. Individual task and total scores were automatically generated by a computer-based algorithm assessing efficiency and accuracy [2]. The FES software and scoring system on the ES simulator is identical to the GIM platforms. Pass status was defined by achieving an overall passing score (binary pass/fail) and determined by applying the standard compensatory cut score per FES policy.

### Statistical analysis

Descriptive statistics were used to summarize examinee demographics, endoscopy experience, and performance measures. The distributions of task-specific and total scores were evaluated for normality using the Shapiro–Wilk test.

#### Simulator platform comparisons

For unadjusted comparisons between simulator platforms, two-sample *t* tests were used for normally distributed continuous outcomes (total score), Mann–Whitney U test was used for non-normally distributed continuous variables (individual task scores), and a chi-squared test was used for categorical variables (pass status). Effect sizes were calculated as *r* for task scores, Cohen’s *d* for total score, and Cohen’s *h* for pass rate.

For adjusted analyses, linear regression was used to model the association between simulator platform and total score (continuous), and logistic regression was used to model pass status (binary). Both regression models were adjusted for age, gender, glove size, and total number of lower endoscopies performed within the last year. A gender-by-simulator platform interaction term was initially included to assess potential effect modification by gender. However, because this interaction was not statistically discernable (*p* > 0.05), it was excluded from the final models.

#### Sensitivity analysis: non-inferiority

Unadjusted comparisons between simulator platforms were also conducted as sensitivity analyses within a non-inferiority framework. For continuous outcomes (individual task scores and total score), a non-inferiority margin was set at Cohen’s *d* = 0.3 to reflect a small-to-moderate difference that would be educationally meaningful from a skill assessment standpoint. Outcome-specific margins were derived by multiplying the pooled standard deviation by 0.3. Non-inferiority of the ES relative to the GIM was assessed using two one-sided tests (TOST) with unequal variances in Stata. For pass rate, a non-inferiority margin of 5 percentage points (Δ = 0.05) was chosen based on educational judgment as the maximum acceptable reduction in pass rate. Noninferiority was assessed using a one-sided *α* = 0.05 and reported as two-sided 90% confidence intervals.

#### Exploratory subgroup analyses

Unadjusted exploratory subgroup analyses of total scores by gender, glove size, and lower endoscopy experience were also conducted. Two-sample *t* tests were used to compare simulator platforms within subgroups and ANOVA with Tukey HSD post-hoc analyses were used for comparisons between subgroups on a single simulator platform.

All analyses were conducted using Stata 19, SPSS, and RStudio (R Foundation for Statistical Computing, Vienna, Austria).

## Results

There was a total of 1,121 FES examinees (ES 337, GIM 784). Of those, 436 examinees (39%) self-reported demographic information on gender, glove size, and endoscopy experience (ES 106, GIM 330). In regard to potential selection bias secondary to examinee exclusion, pass rates between examinees with complete demographic information were comparable to those without demographic information for both simulator platforms (ES 88% vs 88%, *χ*^2^ = 0.02, *p* = 0.88; GIM 83% vs 81%, *χ*^2^ = 0.40, *p* = 0.53) (Table 1).

**Table 1:** Comparison of total scores and pass rates between examinees with complete self-reported demographic data and those without

	Complete demographics	Incomplete demographics	Effect size^a^ (95% CI)	*p*-value
**ES**	*n* = 106	*n* = 231		
Total score (mean, SD)	70 (12)	75 (13)	0.39 (2.0, 7.7)	< 0.001*
Pass rate (%)	88	88	–0.02 (− 0.25, 0.21)	0.88
**GIM**	*n* = 330	*n* = 454		
Total score (mean, SD)	70 (11)	70 (13)	0.35 (–1.3, 2.1)	0.63
Pass rate (%)	83	81	0.05 (− 0.09, 0.19)	0.53

*ES* ENDO Mentor Suite simulator, *GIM* GI Mentor simulator

*p < 0.05 indicates statistically discernable differences; Comparisons made by two-sample* t*-test for total score and chi-square test for pass rate

^a^Effect sizes were calculated as Cohen’s *d* for total score and Cohen’s *h* for pass rate

### Demographic distribution

There were no statistically discernable differences in the level of training, glove size, or upper and lower endoscopy experience between groups (Table 2). There was, however, a small discernable difference in the gender distribution with more women in the ES group (*χ*^2^ = 6.9, *p* = 0.009).

**Table 2:** Comparison of demographic and experience distributions between simulator groups

	ES *n* (%)	GIM *n* (%)	*p*-value
	106	330	
**Level of training**			0.261
Junior (PGY1–3)	45 (42)	120 (36)	
Senior (PGY4+)	61 (58)	210 (64)	
**Gender**			0.009*
Man	41 (38.7)	176 (53.3)	
Woman	65 (61.3)	154 (46.7)	
**Glove size**			0.379
6 or smaller	30 (28.3)	81 (24.5)	
6.5–7	44 (41.5)	125 (37.9)	
7.5 or larger	32 (30.2)	124 (37.6)	
**Upper endoscopy cases (overall)**			0.454
0–24	32 (30.2)	77 (23.3)	
25–49	34 (32.1)	102 (30.9)	
50–99	32 (30.2)	109 (33)	
100–199	6 (5.7)	33 (10)	
200+	2 (1.9)	9 (2.7)	
**Upper endoscopy cases (past year)**			0.546
0–24	57 (53.8)	160 (48.5)	
25–49	36 (34)	112 (33.9)	
50–99	12 (11.3)	50 (15.2)	
100–199	1 (0.9)	8 (2.4)	
200+	0 (0)	0 (0)	
**Lower endoscopy cases (overall)**			0.394
0–24	30 (28.3)	93 (28.2)	
25–49	34 (32.1)	82 (24.8)	
50–99	37 (34.9)	125 (37.9)	
100–199	3 (2.8)	23 (7)	
200 +	2 (1.9)	7 (2.1)	
**Lower endoscopy cases (past year)**			0.780
0–24	58 (54.7)	171 (51.8)	
25–49	35 (33)	105 (31.8)	
50–99	11 (10.4)	47 (14.2)	
100–199	2 (1.9)	7 (2.1)	
200+	2 (1.9)	0 (0)	

*ES* ENDO Mentor Suite simulator, *GIM* GI Mentor simulator

*p < 0.05 indicates statistically discernable differences by chi-square test

### Score distributions

**Fig. 3:**
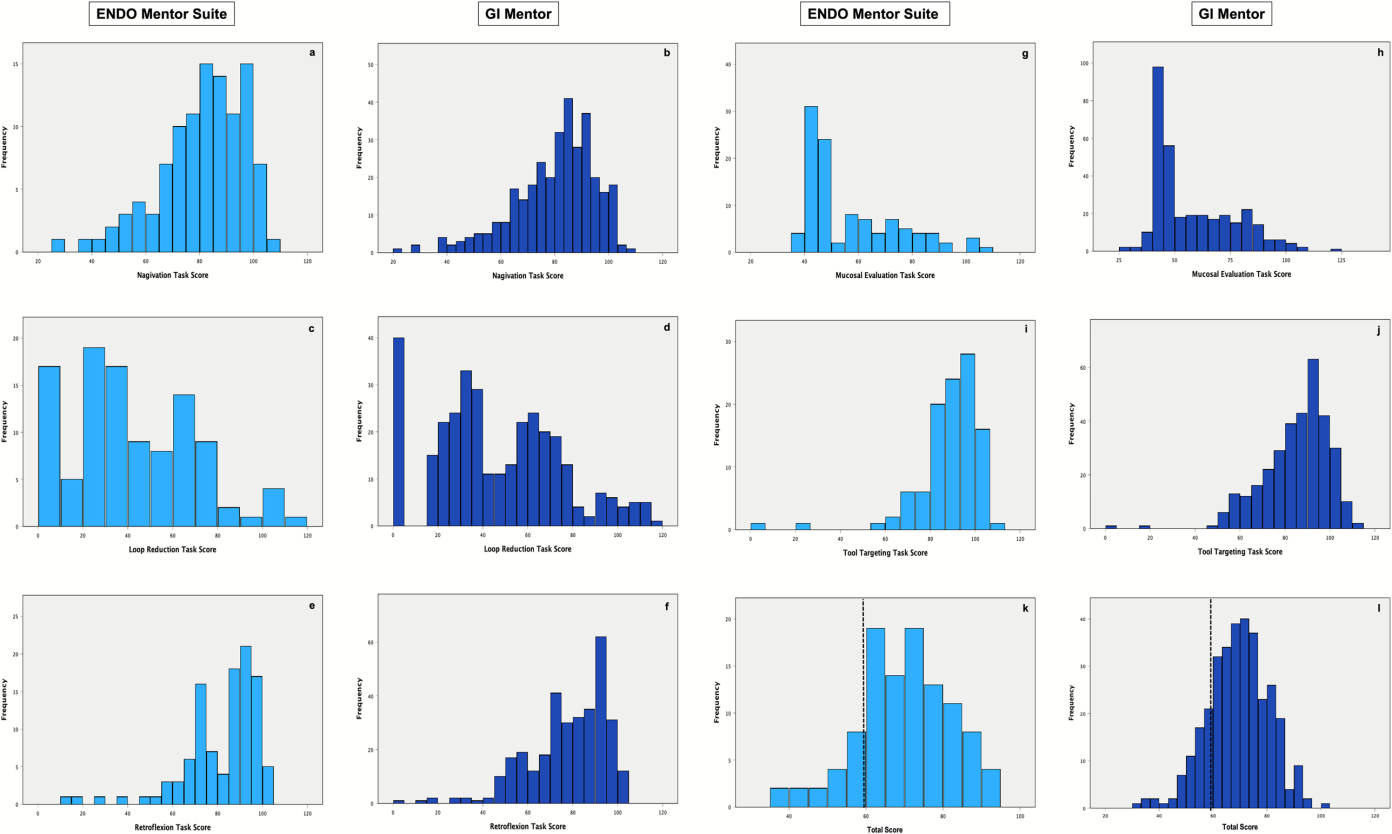
FES task and total score distributions by simulator platform. Dashed line corresponds to passing total score. ENDO Mentor Suite *n* = 106, GI Mentor *n* = 330

A Shapiro–Wilk test of normality revealed a normal distribution for total scores and a non-normal distribution for all individual task scores. Navigation (Fig. 3a, b), Retroflexion (Fig. 3e, f), and Tool Targeting (Fig. 3i, j) demonstrated a right-skewed distribution in scores. Total scores (Fig. 3k, l), however, were more symmetrically distributed.

### Simulator comparisons

In unadjusted analyses for individual tasks, there were statistically discernable differences between ES and GIM groups in performance on Retroflexion and Tool Targeting (Retroflexion 88 vs 81, *r* = 0.12, 95% CI [0.03–0.21], *p* = 0.01; Tool Targeting 91 vs 88, *r* = 0.12, 95% CI [0.03–0.22], *p* = 0.01) (Table 3). For Navigation, Loop Reduction, and Mucosal Evaluation, there were no statistically discernable differences between scores. Total scores were also similar between simulator platforms (70 vs 69, Cohen’s *d* = 0.07, 95% CI [− 0.15, 0.29], *p* = 0.55,), with no statistically discernable differences in pass rates (88% vs 83%, *χ*^2^ = 1.3, Cohen’s *h* = 0.14, 95% CI [− 0.08, 0.36], *p* = 0.25).

**Table 3:** Comparison of FES task scores, total scores, and pass rates between simulator platforms

	ES	GIM	Effect size^a^ (95% CI)	*p*-value
**Task score (median, IQR)**				
Navigation	83 (71–94)	83 (72–92)	–0.04 (–0.14, 0.05)	0.37
Loop reduction	39 (24–62)	41 (27–66)	–0.06 (–0.16, 0.03)	0.19
Retroflexion	88 (73–94)	81 (68–92)	0.12 (0.03–0.21)	0.01*
Mucosal evaluation	48 (45–69)	50 (44–71)	–0.01 (–0.10, 0.09)	0.91
Tool targeting	91 (84–99)	88 (77–95)	0.12 (0.03–0.22)	0.01*
**Total score (mean, SD)**	70 (± 12)	69 (± 11)	0.07 (–0.15, 0.29)	0.55
**Pass rate (%)**	88	83	0.14 (–0.08, 0.36)	0.25

*ES* ENDO Mentor Suite simulator, *GIM* GI Mentor simulator

*p < 0.05 indicates statistically discernable differences; Comparisons made by Mann–Whitney *U* test for task scores, two-sample* t*-test for total score, and chi-square test for pass rate

^a^Effect sizes were calculated as *r* for task scores, Cohen’s *d* for total score, and Cohen’s *h* for pass rate

In adjusted models accounting for age, gender, glove size, and total number of lower endoscopies performed within the last year, simulator platform remained unrelated to total score (*β* = 2.06, 95% CI [− 0.33, 4.47], *p* = 0.09) or pass rate (OR 1.62, 95% CI [0.79, 3.32], *p* = 0.19), and there was no evidence of a gender-by-simulator platform interaction (*p* > 0.05).

### Sensitivity analyses: non-inferiority

In sensitivity analyses, non-inferiority was demonstrated for most individual tasks, total score, and pass status, indicating that performance differences were smaller than the prespecified margins of 0.3 effect size for task scores and 5% for pass rate (Table 4). Two tasks (Retroflexion and Tool Targeting) did not meet criteria for non-inferiority.

**Table 4:** Non-inferiority testing between platforms

Outcome	Difference^a^ (90% CI)	Non-inferiority margin^b^	One-sided *p*-value	Result
**Task**				
Navigation	1.18 (–1.76, 4.13)	4.63	0.027	Non-inferior
Loop reduction	− 4.31 (–9.54, 0.93)	8.54	< 0.001	Non-inferior
Retroflexion	4.18 (1.01, 7.35)	5.21	0.278	Non-inferiority not demonstrated^2^
Mucosal evaluation	− 0.74 (–3.96, 2.48)	5.36	0.001	Non-inferior
Tool targeting	3.49 (0.77, 6.20)	4.42	0.287	Non-inferiority not demonstrated^2^
**Total score**	0.77 (–1.43, 2.97)	3.45	0.023	Non-inferior
**Pass status**	4.7% (2.7%, 12%)	5%	0.005	Non-inferior

Only the cases with complete demographics were used for analysis (*n* = 436)

^a^Mean difference and non-inferiority margin is expressed in points for task score variables and proportion difference for pass/fail. Difference = ES-GIM

^b^Non-inferiority was not demonstrated, indicating that the data are compatible with differences exceeding the prespecified margin

### Exploratory subgroup analyses

#### Within demographic subgroups

Among examinees of the same gender, similar glove sizes (e.g., all with glove size 6.5–7), or similar lower endoscopy experience (e.g., all with 50–99 cases), there were no discernable differences in total scores based on which simulator platform the exam was administered on (Table 5).

#### Between demographic subgroups

*Gender:* Mean total score among men was higher compared to women on both simulator platforms (ES 74 vs 67, *p* = 0.003, GIM 71 vs 67, *p* < 0.001) (Table 5).

Glove Size: Among the GIM examinees, those with glove size 7.5 or larger achieved higher total scores compared to those with glove size 6 or smaller (72 vs 66, *p* < 0.001) (Table 5). This paralleled the results among the ES examinees, in which those with larger glove sizes demonstrated a higher mean total score, yet this difference was not statistically discernable (74 vs 69, *p* = 0.06). There was a small, positive correlation between glove size and total score on both simulator platforms (ES Spearman’s rho = 0.218, *p* = 0.025; GIM Spearman’s rho = 0.196, *p* < 0.001).

Lower Endoscopy Experience: Among the ES examinees, there was no statistically discernable difference in performance secondary to lower endoscopy experience (Table 5). Among the GIM examinees, those with over 200 lower endoscopy cases obtained higher total scores compared to those with fewer cases (vs 0–24 cases: difference in means = 18, *p* < 0.001; vs 25–49 cases: difference in means = 16, *p* = 0.003; vs 50–99 cases: difference in means = 15, *p* = 0.004).

**Table 5:** Comparison of mean total scores between simulator platforms by demographic and experience subgroups

	ES (mean, SD)	GIM (mean, SD)	*p*-value (within subgroups)
**Gender**			
Man	74 (12)	71 (11)	0.18
Woman	67 (12)	67 (11)	0.64
* p*-value (between subgroups)	0.003*	< 0.001*	
**Glove size**			
6 or smaller	69 (9)	66 (11)^a^	0.24
6.5–7	68 (13)	69 (11)	0.59
7.5 or larger	74 (13)	72 (12)^b^	0.27
* p*-value (between subgroups)	0.06	< 0.001*	
**Lower endoscopy** **cases (overall)**			
0–24	67 (13)	67 (11)^a^	0.87
25–49	71 (11)	69 (10)^a^	0.45
50–99	71 (12)	70 (11)^a^	0.39
100–199	73 (8)	73 (12)	0.97
200 +	82 (12)	85 (11)^b^	0.78
* p*-value (between subgroups)	0.33	< 0.001*	

*ES* ENDO Mentor Suite simulator, *GIM* GI Mentor simulator

*p < 0.05 indicates statistically discernable differences by independent *t-*test within subgroups and ANOVA between subgroups

^a,b^For between subgroups, different superscripts indicate statistically discernable differences on post-hoc analysis

## Discussion

This study demonstrates the ES does not introduce significant construct irrelevant variance for its use in the FES manual skills examination. With the exception of Mucosal Evaluation, the distributions of FES task and total scores on both the ES and GIM appear consistent with prior studies [7]. While all task scores appear similar, performance on Retroflexion and Tool Targeting was discernably higher on the ES compared to the GIM. Nonetheless, scores for both tasks shared overlapping interquartile ranges, and the effect size was small. Additionally, sensitivity analyses yielded a similar pattern whereby performance on the ES met criteria for non-inferiority for Navigation, Loop Reduction, Mucosal Evaluation, total score, and pass status. Potential educationally-meaningful differences could not be definitively ruled out for Retroflexion and Tool Targeting. Consistent with the compensatory nature of the exam, performance differences at the task level did not translate into differences in total scores or pass rates. These findings of similar FES performance between the ES and GIM are consistent with the first round results from the preliminary randomized control trial. Furthermore, the previously observed scoring discrepancies are not redemonstrated in this larger data set.

Within demographic subgroups (e.g., all examinees with small glove sizes), overall FES performance on the ES was similar to that on the GIM. This demonstrates that the ES does not introduce additional biases among individuals with similar characteristics. However, exploratory analyses between subgroups (e.g., men compared to women on the ES) revealed discernable differences in performance for gender, glove size, and endoscopy experience, consistent with findings in prior studies [3, 7].

With regard to gender, the average total score among men in this sample was higher on both the ES and GIM compared to women. While causal mechanisms were not examined in this study, gender differences in endoscopy have been previously reported and are primarily attributed to greater visuospatial aptitude seen among most men, a discrepancy that may be equalized through visuospatial training [7–10]. Yet, despite there being significantly more women in the ES group, this did not appear to negatively impact the total score. Additionally, GIM examinees with larger glove sizes and greater lower endoscopy experience performed better than those with smaller glove sizes and less lower endoscopy experience, respectively. These differences were not statistically discernable among ES examinees. However, given similar trends in the numerical distribution of total scores, the absence of statistical discernability may be secondary to lower power in the ES group.

A notable limitation of this study is that analyses involving demographic covariates were restricted to examinees with self-reported demographic data, resulting in the exclusion of more than half of the examinees in each simulator group. This complete-case approach reduces the effective sample size and therefore decreases statistical power, increasing the risk of type II error, and may introduce selection bias if examinees who declined to report demographics differ systematically from those who did. Multiple imputation was considered but judged inappropriate because demographic data were mostly either fully complete or entirely missing per examinee, and insufficient auxiliary variables were available to support a robust imputation model. This limitation highlights the need for stronger quality control processes for demographic data collection at the time of the manual skills exam. Efforts to optimize this process are currently ongoing.

In summary, this analysis reveals that the new ES platform produces similar FES performance results as the original GIM and does not introduce construct irrelevant variance. As the ES continues to become more widely incorporated into accredited FES testing centers, it will be imperative to improve collection of quality data in order to continue conducting validity investigations.

## References

[1] Fundamentals of Endoscopic Surgery (n.d.) Testing information. Available at: https://www.fesprogram.org/testing-information/. Accessed 25 Feb 2025

[2] Vassiliou MC, Dunkin BJ, Fried GM, Mellinger JD, Trus T, Kaneva P, Lyons C, Korndorffer JR Jr., Ujiki M, Velanovich V, Kochman ML, Tsuda S, Martinez J, Scott DJ, Korus G, Park A, Marks JM (2014) Fundamentals of endoscopic surgery: creation and validation of the hands-on test. Surg Endosc 28:704–711 24253562 10.1007/s00464-013-3298-4

[3] Mueller CL, Kaneva P, Fried GM, Feldman LS, Vassiliou MC (2014) Colonoscopy performance correlates with scores on the FES™ manual skills test. Surg Endosc 28:3081–3085 24902817 10.1007/s00464-014-3583-x

[4] Surgical Science (n.d.) Surgical Science ENDO Suite. Available at: https://surgicalscience.com/endo-suite-2/. Accessed 25 Feb 2025

[5] American Educational Research Association, American Psychological Association, National Council on Measurement in Education (2014) Standards for educational and psychological testing. Washington, DC: American Educational Research Association

[6] Miller PM, Martinez J, Pauli E, Mischna J, Ritter EM (2024) Validity evidence for the use of ENDO mentor suite for the fundamentals of endoscopic surgery skills examination. Presented at Society of American Gastrointestinal and Endoscopic Surgeons Annual Meeting, Cleveland, Ohio

[7] Lineberry M, Matthew Ritter E (2017) Psychometric properties of the fundamentals of endoscopic surgery (FES) skills examination. Surg Endosc 31:5219–5227 28493166 10.1007/s00464-017-5590-1

[8] Doster D, Park YS, Tekian A, Collings AT, Thomas CM, Stefanidis D, Ritter EM (2022) Why the gap? Analyzing the etiology of gender-based performance discrepancy in laparo-endoscopic skills. Masters thesis, University of Illinois Chicago

[9] Enochsson L, Isaksson B, Tour R, Kjellin A, Hedman L, Wredmark T, Tsai-Fellander L (2004) Visuospatial skills and computer game experience influence the performance of virtual endoscopy. J Gastrointest Surg 8:876–882 15531242 10.1016/j.gassur.2004.06.015

[10] Kass SJ, Ahlers RH, Dugger M (1998) Eliminating gender differences through practice in an applied visual spatial task. Hum Perform 11:337–349

